# Increased disease activity in early arthritis patients with anti-carbamylated protein antibodies

**DOI:** 10.1038/s41598-021-89502-y

**Published:** 2021-05-11

**Authors:** Cristina Regueiro, Laura Nuño, Ana Triguero-Martinez, Ana M. Ortiz, Alejandro Villalba, María Dolores Bóveda, Ana Martínez-Feito, Carmen Conde, Alejandro Balsa, Isidoro González-Alvaro, Antonio Gonzalez

**Affiliations:** 1 Experimental and Observational Rheumatology and Rheumatology Unit, Instituto de Investigacion Sanitaria - Hospital Clínico Universitario de Santiago (IDIS), 15706 Santiago de Compostela, Spain; 2 Rheumatology Department, Instituto de Investigación Hospital Universitario la Paz (IDIPAZ), 28046 Madrid, Spain; 3 Rheumatology Department, Hospital Universitario de la Princesa, Instituto de Investigación Sanitaria la Princesa (IIS-lP), 28006 Madrid, Spain; 4 Unit of Diagnosis and Treatment of Congenital Metabolic Diseases, Department of Paediatrics, Instituto de Investigación Sanitaria - Hospital Clínico Universitario de Santiago, 15706 Santiago de Compostela, Spain; 5 Immuno-Rheumatology Department, Instituto de Investigación Hospital Universitario La Paz (IDIPAZ), 28046 Madrid, Spain

**Keywords:** Rheumatoid arthritis, Prognostic markers

## Abstract

The initial management of rheumatoid arthritis (RA) has a high impact on disease prognosis. Therefore, we need to select the most appropriate treatment as soon as possible. This goal requires biomarkers of disease severity and prognosis. One such biomarker may be the presence of anti-carbamylated protein antibodies (ACarPA) because it is associated with adverse long term outcomes as radiographic damage and mortality. Here, we have assessed the ACarPA as short-term prognostic biomarkers. The study was conducted in 978 prospective early arthritis (EA) patients that were followed for two years. Our results show the association of ACarPA with increased levels of all the disease activity measures in the first visit after arthritis onset. However, the associations were more significant with the high levels in local measures of inflammation and physician assessment than with the increases in systemic inflammation and patient-reported outcomes. More notably, disease activity was persistently increased in the ACarPA positive patients during the two years of follow-up. These differences were significant even after accounting for the presence of other RA autoantibodies. Therefore, the ACarPA could be considered short-term prognostic biomarkers of increased disease activity in the EA patients.

## Introduction

Rheumatoid arthritis (RA) is a chronic autoimmune disease characterized by inflammation at multiple joints with a symmetrical and peripheral distribution^1^. Symptoms and signs include pain, morning stiffness, joint swelling, fatigue and functional disability. Further, if RA is untreated, it will lead to bone erosions, deformities, significant handicap, and life shortening. Effective treatments are available that make these adverse outcomes less frequent. However, there are still many areas of RA management requiring improvement^1^. The early clinical phase is particularly challenging because control of the inflammation at this stage is critical for the prognosis^2^. Unfortunately, there is not sufficient information to decide on the type and level of treatment. The few accepted features associated with a poor prognosis provide limited information, but they are recommended for patient assessment even if a definite diagnosis cannot be reached and the patient has early undifferentiated arthritis^3–5^. Only three of them could be present at the first visit: high disease activity, erosions, and any of two RA autoantibodies. High disease activity is commonly defined as a Disease Activity Score 28-Erythrocyte Sedimentation Rate (DAS28-ESR) greater than 5.1^6^. DAS28-ESR is a composite index combining the number of swollen and tender joints, the ESR reflecting the acute phase response, and the patient reported general health^6^. In turn, the two autoantibodies known to be associated with poor prognosis are rheumatoid factor (RF), IgM antibodies against the Fc of IgG immunoglobulins^7^, and the anti-citrullinated peptides antibodies (anti-CCP). The anti-CCP antibodies recognize citrullinated proteins, which have undergone citrullination, an enzyme-mediated posttranslational modification consisting in the change of arginine for citrulline^8^.

The search for other prognostic biomarkers is still ongoing^9–12^. One possibility is that other RA autoantibodies could complement the information provided by RF and anti-CCP. The most studied are the anti-carbamylated protein antibodies (ACarPA)^12^. The carbamylated proteins have undergone a posttranslational modification changing lysine to homocitrulline, which is structurally related to citrulline. These antibodies have favorable characteristics as a potential biomarker in EA: their presence and specificity for RA are widely reproducible between laboratories and they are already present at arthritis onset^13–16^. Also, they identify a subgroup of RA patients with adverse outcomes in the long-term. In effect, they are associated with the progression of radiographic damage^12,15–18^, the loss of bone mineral density^19^, the presence of interstitial lung disease^20^, and increased mortality^21^. All these associations have been statistically independent of the presence of anti-CCP or RF indicating the ACarPA are specific biomarkers of poor prognosis.

Regarding the short-term outcomes, the presence of ACarPA has been associated with higher disease activity (assessed with DAS28-ESR) at arthritis onset in several large EA cohorts^18,22–24^. The EA cohorts are recruited in specialized clinics soon after the onset of the clinical manifestations and followed with standardized protocols^25,26^. The patients in the EA clinics show improved outcomes and the EA cohorts provide very useful information about the initial disease characteristics. Despite the ACarPA consistent association with high DAS28-ESR, they are not yet recognized as useful biomarkers in EA patients. Therefore, we have addressed the association of ACarPA with disease activity trying to clarify the value of ACarPA as short-term prognostic biomarkers at arthritis onset.

## Material and methods

### Patients and samples

Patients were recruited in prospective EA clinics at Hospital Universitario La Paz (January 1993 to December 2013)^27^ and Hospital Universitario La Princesa (July 2001 to December 2014)^28^. The inclusion criteria were the presence of ≥ 2 swollen joints (≥ 1 swollen joint from 2010 at La Princesa following the implementation of the 2010 ACR/EULAR RA classification criteria^29^, n = 154 patients) for < 1 year without any previous disease-modifying anti-rheumatic drugs (DMARD). The patients were examined at 6, 12, and 24 months. Only EA patients that have completed the 2 years of follow-up and with available serum and DAS28-ESR at baseline were analyzed here (n = 978). Determinations of ACarPA in the baseline sera were performed after finishing the follow-up and therefore with total independence of the patient management.

The EA clinics and the sample collections were approved by the La Paz University Hospital and the Hospital Universitario La Princesa (Ref. PI-518) Research Ethics Committees. The study was approved at the Hospital Clínico Universitario de Santiago (Ref. 2014/387 and 2017/514). All participating subjects gave their written informed consent and all protocols and methods were conducted according to the Declaration of Helsinki and the Belmont Report and the Spanish regulations.

### Measures of disease activity

Seven core disease activity measures were analyzed, including the four combined in the DAS28-ESR, swollen joint count (SJC), tender joint count (TJC), patient global assessment (PtGA), and erythrocyte sedimentation rate (ESR)^6^; and three others, the physician global assessment (PhGA), and the patient assessment of physical function (HAQ) and the pain visual analog scale (VAS)^30^. The DAS28-ESR components were considered with the corresponding transformations (i.e., the squared root of SJC and TJC, and the natural logarithm of the ESR). Two other composite measures of disease activity, the Clinical Disease Activity Index (CDAI)^31^, and the Hospital Universitario La Princesa Index (HUPI)^32^, were assessed. The levels of DAS28-ESR, HAQ, and pain VAS were considered quantitatively for the analyses and according to published strata for the description of results^30–32^. The strata for DAS28-ESR were: remission ≤ 2.6, low disease activity > 2.6 and ≤ 3.2, moderate disease activity > 3.2 and ≤ 5.1 and high disease activity > 5.1^33^. The HAQ levels were: scores of 0 to 1 represent mild to moderate difficulty, 1 to 2 moderate to severe disability, and 2 to 3 severe to very severe disability^34^. Finally, the qualitative description of pain VAS used the thresholds: 0 to 4 mm, no pain; 5 to 44 mm, mild pain; 45 to 74 mm, moderate pain; and 75 to 100 mm, severe pain^35^. In addition, the described thresholds for minimal clinically important differences (MCID) or minimal clinically important improvements (MCII) were indistinctly considered for interpretation of the results depending on their availability (Supplementary Table S1)^36–40^.

### Autoantibodies

All the autoantibody data were available from a previous study^41^. Briefly, the ACarPA were assayed against in vitro carbamylated fetal calf serum FCS (F-7524, Sigma-Aldrich) as described^12,15^. In vitro carbamylation of proteins was performed by incubating 4 mg/mL FCS with 1 M KCNO at 37 °C 15 h. The carbamylation efficiency and percentage were corroborated by HPLC (Supplementary Fig. 1). The IgG ACarPA were quantified by ELISA in separate plates coated with FCS and with carbamylated FCS. The reactivity to unmodified FCS was subtracted from the reactivity to carbamylated FCS. The results reproducibility was assessed by running all samples in duplicate and by including a low titer sample in all plates (mean intra-assay CV = 7.0 and 6.1% against unmodified and carbamylated FCS, respectively, and mean inter-assay CV = 8.7 and 8.8% against unmodified and carbamylated FCS, respectively). A standard curve, made with serial dilutions from a pool of positive sera, was used to measure antibody levels in arbitrary units. The cut-off for positivity was set at 98% specificity level obtained in the 208 healthy controls (see Supplementary Fig. S2 for the comparison of healthy controls with EA patients). The RF and anti-CCP antibodies were determined as part of the routine care. The IgM-RF was determined by nephelometry and the anti-CCP were tested as anti-cyclic citrullinated peptide (anti-CCP) antibodies by ELISA.

### Statistical analysis

The activity measures association with the ACarPA status (positive/negative) or levels (negative/low positive/high positive) was evaluated with main effects general linear regression. The analysis of the fraction with high disease activity or in remission was done with logistic regression. In all cases, the reported parameters are the standardized slopes (β) with their standard error (SE) and *p* value, and the estimated marginal means (EMM) with their 95% CI. The standardized slopes (β) allow for comparison of the activity measures association because they are independent of the measurement scale. Also, the standardized slopes (β) can be interpreted as the factor-specific regression coefficients, which reflect the fraction of the variance accounted for by each factor. An EMM is the activity measure mean in the subgroup (ACarPA^+^ or ACarPA^−^) adjusted for the factors and covariates in the regression model. Accordingly, the differences between the activity measures in ACarPA^+^ and ACarPA^−^ patients were calculated as differences between the respective EMM, and the percentage of change was calculated as ACarPA^+^ EMM − ACarPA^−^ EMM / ACarPA^−^ EMM × 100. The three transformed DAS28-ESR components (sqrSJC, sqrTJC, and lnESR) were considered in the transformed form for the analyses because these transformations approach their distributions to the normal distribution^6^. However, the EMM and CI corresponding to these three DAS28-ESR components were back-transformed for reporting. Sex, age, and the specific EA clinic were included as confounding variables in the analyses. Sex and cohort were included as factors and age as a covariate in the general linear regression models. These three variables together were considered the basic adjustment and labeled “basic” in the tables. In additional analyses, we verified that smoking and time since symptoms onset did not change the reported results. These analyses were not included because of the larger fraction of missing data for these two variables (the missing data for all variables are detailed in Table 1 footnote b). Additional multivariate analyses including the anti-CCP status or levels, the RF status, or the final patient classification were also performed where indicated. Also, we assessed the association of the activity measures with the ACarPA levels using Spearman rank regression. All the previous analyses were done with Statistica 7.0 (StatSoft, Tulsa, OK). In addition, the longitudinal data of follow-up on DAS28-ESR and HAQ were analyzed for association with the ACarPA status. Two types of analysis were used: analysis of the cases with complete data at the four follow-up times, and analysis of all the available follow-up data with mixed-effects pattern-mixture models for repeated measures^42–44^. The latter procedure assesses the data for patterns of missing not at random (MNAR) data and controls for them in linear mixed-effects models. In our case, the MNAR patterns, the confounding factors (cohort, sex, age, anti-CCP status, and RF status), and the times of follow-up were considered as fixed effects in the linear mixed models. The models were completed by considering the patients as the random effects. This procedure allowed for individual DAS28-ESR or HAQ trajectories defined by the available information for each patient. These analyses were conducted with Jamovi 1.6 implementing the GAMj module^45^.

**Table 1:** Clinical and serological features of the EA patients.

Feature	EA^a^ patients,
	n = 978
Women, n (%)	750 (76.7)
Years, median (IQR)^b^	52.2 (40.1–65.5)
Weeks since onset, median (IQR)^b^	16 (8–28)
Calendar year of onset, median (IQR)	2008 (2004–11)
Smokers, n (%)	412 (42.1)
Classified as RA at 2 years^c^, n (%)	505 (51.6)
RF positive, n (%)	416 (42.5)
CCP positive, n (%)	376 (38.4)
ACarPA positive, n (%)	275 (28.1)
DAS28-ESR, median (IQR)	
0 months	4.4 (3.3–5.6)
6 months^b^	3.0 (2.3–4.0)
12 months^b^	3.1 (2.2–3.9)
24 months^b^	2.7 (2.1–3.7)
DAS28-ESR components at baseline, median (IQR)	
VAS pain	47 (24–69)
Patient global assessment	4.5 (2.5–6.3)
Physician global assessment	3.5 (2.0–5.0)
Tender joint count	4 (1–9)
Swollen joint count	3 (1–7)
ESR	24 (13–44)
HAQ, median (IQR)	
0 months	1.00 (0.50–1.63)
6 months^b^	0.38 (0.00–0.88)
12 months^b^	0.38 (0.00–0.88)
24 months^b^	0.38 (0.00–0.88)
Initial treatment^b^	
Corticosteroids, n (%)	223 (41.7)
Methotrexate, n (%)	327 (61.1)
MTX dose, median (IQR)	15 (10–15)
Other csDMARDs, n (%)	71 (13.3)
Any of the drugs^d^, n (%)	445 (83.2)
Unmodified in the first 6 months, n (%)	212 (39.6)

^a^EA = early arthritis; IQR = interquartile range; RF = rheumatoid factor; CCP = anti-cyclic citrullinated peptide antibodies; ACarPA = anti-carbamylated protein antibodies; DAS28-ESR = disease activity score 28 joints; VAS = visual analogic scale; ESR = erythrocyte sedimentation rate; HAQ = Health Assessment Questionnaire; MTX = methotrexate; cDMARD = conventional synthetic Disease-Modifying Anti-Rheumatic Drug.

^b^Incomplete data were available for age (968 patients), weeks since onset (942), smoking (915), DAS28-ESR at 6 months (637), 12 months (519) and 24 months (504); HAQ at baseline (949), 6 months (665), 12 months (617) and 24 months (654); and for initial treatment (535).

^c^Classified according to the 1987 ACR classification criteria.

^d^The other drugs = corticosteroids or MTX or other csDMARD.

## Results

### Increased disease activity in the ACarPA^+^ EA patients

The characteristics of the EA patients (Table 1 and Supplementary Table S2) have already been described^41^. The ACarPA positive patients were 28.1%, which is lower than the fraction bearing either anti-CCP or RF (38.4 and 42.5%, respectively. See supplementary Fig. S3 for the autoantibodies distribution). Median disease activity was moderate at onset (DAS28-ESR = 4.4). It became low in the next visit (DAS28-ESR = 3.0) and remained low thereafter. This reduction of the disease activity was also reflected in the fraction of EA patients with high disease activity (i.e., DAS28-ESR > 5.1): from 33.8% in the first visit to ≤ 9.2% in subsequent visits.

The ACarPA^+^ patients showed higher disease activity than the ACarPA^−^ patients at the first visit (Table 2). The increase was significant also in the analysis adjusted for confounding variables and accounting for the presence of anti-CCP and RF (Table 2). Notably, the differences between the ACarPA^+^ and ACarPA^−^ patients were only modestly reduced in the multivariate comparisons. Specifically, the mean increase was 0.64 (4.30 *vs.* 4.94) in the univariate analysis and 0.45 (4.26 *vs.* 4.71) in the full multivariate model. The latter was particularly relevant given the frequent concordance of the RA autoantibodies in the EA patients (Supplementary Fig. S3). None of the two values, the univariate or the multivariate difference, was over 1.2, the reported MCII for DAS28-ESR^36,38^.

**Table 2:** Increased composite disease activity measures in the ACarPA^+^ than in the ACarPA^−^ patients at baseline^a^.

Measure/Adjusted	β^b^	SE	*p* value	ACarPA^+^ EMM	ACarPA^−^ EMM
DAS28-ESR					
no^c^	0.18	0.03	8.9 × 10^–9^	4.94 (4.76–5.12)	4.30 (4.12–4.42)
basic^c^	0.18	0.03	9.1 × 10^–9^	4.79 (4.60–4.97)	4.17 (4.04–4.29)
basic + CCP^c^	0.13	0.03	7.1 × 10^–5^	4.72 (4.53–4.91)	4.26 (4.12–4.39)
basic + RF^c^	0.15	0.03	1.3 × 10^–5^	4.73 (4.54–4.93)	4.22 (4.09–4.35)
basic + CCP & RF^c^	0.13	0.03	1.3 × 10^–4^	4.71 (4.52–4.91)	4.26 (4.12–4.40)
CDAI					
basic + CCP & RF^c,d^	0.11	0.04	1.5 × 10^–3^	21.7 (19.9–23.5)	18.3 (17.0–19.5)
HUPI					
basic + CCP & RF^c^	0.12	0.03	4.3 × 10^–4^	7.81 (7.40–8.22)	6.92 (6.63–7.21)

^a^Data were available from 978 patients in the first-row analysis and 99% of them in the other DAS28-ESR analyses; from 902 patients in the CDAI analysis and from 974 patients in the HUPI analysis.

^b^The slope coefficients (β) with their standard errors (SE) and *p* values and the estimated marginal means (EMM) with their 95% CI are presented.

^c^The ACarPA^+^ and ACarPA^−^ EA patients were compared without adjustment or with the basic adjustment for sex, age, and the specific cohort (labeled “basic”), or with adjustment including also the presence of anti-CCP, RF, or both autoantibodies.

^d^Similar results were obtained after transforming the CDAI values to conform with the normal distribution: β = 0.11, SE = 0.04, *p* = 2.1 × 10^–3^, ACarPA^+^ EMM = 18.9 (17.1–20.8), ACarPA^−^ EMM = 15.6 (14.5–16.9).

Two other composite measures of disease activity were analyzed, the CDAI and the HUPI. They showed very significant increases in the ACarPA^+^ patients at the first visit. The difference remained significant in the full multivariate analysis adjusting for the presence of RF and anti-CCP, which are the results reported in Table 2.

### Local component of the increased DAS28-ESR in the ACarPA^+^ patients

The four components of the DAS28-ESR were increased in the ACarPA^+^ patients at the first visit (Table 3). The increase was more marked in the SJC and TJC (60%, 2.99 *vs.* 4.80, and 58%, 3.65 *vs.* 5.76, respectively) than in the other two components, PtGA and ESR (12%, 43.17 *vs.* 48.21, and 27%, 21.33 *vs.* 27.11, respectively). Moreover, the increase in SJC and TJC were independent of the presence of anti-CCP and RF as they remained significantly different in the full multivariate analysis (Table 3). In contrast, the difference in ESR was no longer significant in the fully adjusted analysis (*p* = 0.067) and the difference in PtGA was borderline (*p*= 0.049). The difference in PtGA did not reach the described MCII^36,37^, whereas no MCII has been described for the other DAS28-ESR components.

**Table 3:** DAS28-ESR components in the ACarPA^+^ patients relative to the ACarPA^−^ EA patients at baseline^a^.

Component/Adjustment	β^b^	SE	*p* value	ACarPA^+^ EMM	ACarPA^−^ EMM
Swollen Joint Count					
no^c^	0.18	0.03	3.02 × 10^–8^	4.80 (4.20–5.43)	2.99 (2.69–3.28)
basic^c^	0.18	0.03	1.28 × 10^–8^	4.80 (4.20–5.43)	2.96 (2.66–3.31)
basic + CCP^c^	0.12	0.03	0.0004	4.49 (3.92–5.15)	3.28 (2.92–3.69)
basic + RF^c^	0.14	0.03	0.00005	4.58 (3.96–5.24)	3.17 (2.79–3.53)
basic + CCP & RF^c^	0.12	0.03	0.0008	4.49 (3.88–5.15)	3.31 (2.92–3.69)
Tender Joint Count					
no^c^	0.16	0.03	1.01 × 10^–6^	5.76 (4.97–6.60)	3.65 (3.24–4.04)
basic^c^	0.14	0.03	4.25 × 10^–6^	5.11 (4.37–5.95)	3.28 (2.86–3.69)
basic + CCP^c^	0.13	0.03	0.0002	5.02 (4.24–5.86)	3.39 (2.96–3.88)
basic + RF^c^	0.12	0.03	0.0003	4.97 (4.20–5.81)	3.39 (2.96–3.88)
basic + CCP & RF^c^	0.12	0.03	0.0006	4.97 (4.20–5.81)	3.42 (2.96–3.92)
Patient Global Assessment					
no^c^	0.09	0.03	0.006	48.21 (45.18–51.25)	43.17 (41.27–45.07)
basic^c^	0.08	0.03	0.01	47.0 (43.79–50.20)	43.34 (40.22–44.46)
basic + CCP^c^	0.06	0.03	0.07	46.54 (43.27–49.80)	42.97 (40.68–45.27)
basic + RF^c^	0.08	0.03	0.02	46.97 (43.67–50.27)	42.37 (40.10–44.64)
basic + CCP & RF^c^	0.07	0.04	0.049	46.78 (43.47–50.08)	42.81 (40.49–45.13)
Erythrocyte Sedimentation Rate					
no^c^	0.12	0.03	0.0001	27.11 (24.53–29.96)	21.33 (20.09–22.87)
basic^c^	0.12	0.03	0.00006	24.78 (22.20–27.39)	19.49 (18.54–20.91)
basic + CCP^c^	0.07	0.03	0.044	23.57 (21.33–26.31)	20.70 (19.30–22.42)
basic + RF^c^	0.08	0.03	0.015	23.81 (21.33–26.58)	20.29 (18.92–21.98)
basic + CCP & RF^c^	0.06	0.03	0.067	23.57 (21.12–26.05)	20.91 (19.30–22.42)

^a^Data from 978 EA patients were analyzed in the first row of each component and 99% of them in the remaining rows.

^b^Results are presented as in the Table 2.

^c^The analyses were performed as in the Table 2 except that sqr(SJC), sqr(TJC) and ln(ESR) transformations were used in the general linear regression. EMM and their 95% CI corresponding to these three measures were back-transformed for reporting.

### Additional disease activity measures

Overall patient disability evaluated with the HAQ was moderate at the first visit (HAQ = 1.00, Table 1) and low at all subsequent visits (HAQ = 0.38, Table 1). This pattern was also reflected in the low fraction of EA patients reporting severe disability (i.e., 15.8% with HAQ ≥ 2). Within this pattern of modest disability, the ACarPA^+^ patients showed increased disability at the first visit (Table 4). The mean difference (14.5%, 1.03 *vs.* 1.18) was maintained in the univariate and multivariate analyses, including the analysis accounting for the presence of anti-CCP and RF (14.1%, 0.99 *vs.* 1.13, Table 4). However, the increase in the ACarPA^+^ patients (0.15 point, 1.03 *vs.* 1.18) was not larger than the published MCII^36,37^.

**Table 4:** Increased disability, pain and physician global assessment in the ACarPA^+^ patients at baseline^a^.

Measure/Adjustment	β^b^	SE	*p* value	ACarPA^+^ EMM	ACarPA^−^ EMM
Disability index (HAQ)					
no^c^	0.09	0.03	0.008	1.18 (1.09–1.27)	1.03 (0.98–1.09)
basic^c^	0.08	0.03	0.008	1.13 (1.03–1.22)	0.98 (0.92–1.05)
basic + CCP^c^	0.07	0.03	0.04	1.12 (1.02–1.21)	1.00 (0.93–1.07)
basic + RF^c^	0.09	0.03	0.008	1.13 (1.04–1.23)	0.98 (0.91–1.05)
basic + CCP & RF^c^	0.08	0.04	0.02	1.13 (1.03–1.23)	0.99 (0.92–1.06)
Pain (VAS)					
no^c^	0.07	0.03	0.021	49.66 (46.43–52.89)	45.18 (43.16–47.20)
basic^c^	0.07	0.03	0.041	47.72 (44.31–51.13)	43.79 (41.53–46.04)
basic + CCP^c^	0.05	0.03	0.151	47.33 (43.86–50.81)	44.33 (41.90–46.77)
basic + RF^c^	0.06	0.03	0.065	47.69 (44.19–51.20)	43.82 (41.41–46.23)
basic + CCP & RF^c^	0.06	0.04	0.117	47.54 (44.02–51.05)	44.19 (41.72–46.65)
Physician Global Assessment					
no^c^	0.18	0.03	4.12 × 10^–8^	42.63 (39.92–45.34)	33.58 (31.86–35.30)
basic^c^	0.18	0.03	5.47 × 10^–8^	42.46 (39.59–45.32)	33.59 (31.66–35.51)
basic + CCP^c^	0.14	0.04	9.82 × 10^–5^	41.60 (38.70–44.50)	34.79 (32.74–36.85)
basic + RF^c^	0.17	0.04	2.13 × 10^–6^	42.21 (39.27–45.16)	33.83 (31.79–35.87)
basic + CCP & RF^c^	0.15	0.04	3.98 × 10^–5^	41.92 (38.99–44.85)	34.59 (32.52–36.67)

^a^Data from 949, 966 and 903 patients were available for the first-row analysis of the HAQ, VAS and PhGA analyses, respectively. The remaining analyses counted with 99% of these patients.

^b^The results are presented as in the Table 2.

^c^The analyses were performed as in the Table 2.

The median pain VAS was moderate at baseline (Table 1), with only 17.3% of the patients reporting severe pain (i.e., ≥ 75 mm). Even so, the values were significantly higher in the ACarPA^+^ than in the ACarPA^−^ patients (Table 4). However, the difference was small (9.0% or 4.5 mm, 45.18 *vs.* 49.66) and only significant when no other antibody was included in the analysis. In effect, the multivariate analyses including either anti-CCP, RF, or the two antibodies did not show a significant difference (Table 4). Also, the difference did not reach the published MCII^36,37^.

The PhGA was also significantly larger in the ACarPA^+^ than in the ACarPA^−^ patients (Table 4). The difference was more notable for this outcome than for the patient-reported outcomes (27.0% or 9.1 mm, 33.58 *vs.* 42.63). It was significant in all the analyses including the full multivariate model.

### Additional analyses of the first visit activity measures associations with the ACarPA

We completed our evaluation of the association of the activity measures with the ACarPA by considering the ACarPA and ACPA levels, the potential interactions between the autoantibodies, and the final patients’ classification. None of these analyses changed substantially the results we have already described, although some insight was obtained by considering the ACarPA levels.

**Figure 1:**
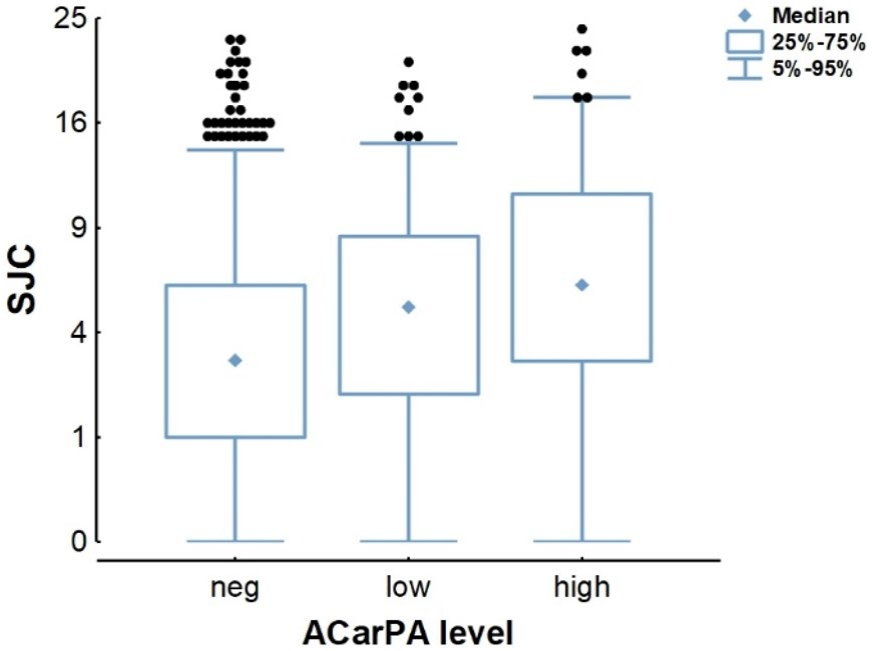
Increased SJC with high ACarPA titers. Box plots showing the SJC stratified in ACarPA^−^ patients (n = 702) and ACarPA^+^ patients with either low (n = 180) or high titers (n = 96). The median (diamonds), interquartile range (boxes), and 5–95% range (whiskers) are shown.

The ACarPA levels were considered in two sets of analyses. The first analyses, considering the rank correlation with the ACarPA levels, did not produce any new information (Supplementary Table S3). In effect, the three patient-reported outcomes showed either weak (*p* = 0.022 and 0.042 for PtGA and HAQ, respectively) or non-significant association (*p* = 0.056 for Pain VAS) as with the ACarPA status. Also, all the other measures showed clearly significant results resembling the associations with the ACarPA status (all *p* < 5.7 × 10^–5^, Supplementary Table S3). The new insight was obtained after stratifying the patients into three groups according to the ACarPA levels (negative/low positive/high positive, Table 5). One of the measures, SJC, showed a significantly larger association with the high titers of ACarPA than with the low titers in the unadjusted analyses (*p* = 0.030). The difference in SJC between the high and low ACarPA patients (1.4, 4.3 *vs.* 5.7) was similar to the difference between the low and ACarPA^−^ patients (1.3, 3.0 *vs.* 4.3, Fig. 1). The difference did not reach statistical significance in the full multivariate analysis (*p* = 0.063, supplementary Table S4). None of the other activity measures showed significant differences between patients with high and low titers among the ACarPA^+^ patients (Table 5).

**Table 5:** Stronger association of SJC with high than with low levels of ACarPA^a^.

Measure	1st level ^b^	2nd level	β	SE	*p* value
DAS28-ESR	neg	low	0.15	0.03	1.4 × 10^–5^
	neg	high	0.16	0.03	4.0 × 10^–6^
	low	high	0.06	0.06	0.29
CDAI	neg	low	0.13	0.03	2.6 × 10^–4^
	neg	high	0.15	0.04	6.3 × 10^–5^
	low	high	0.06	0.06	0.36
HUPI	neg	low	0.15	0.03	1.1 × 10^–5^
	neg	high	0.15	0.04	1.9 × 10^–5^
	low	high	0.05	0.06	0.43
SJC	neg	low	0.12	0.03	2.9 × 10^–4^
	neg	high	0.18	0.03	1.8 × 10^–7^
	low	high	0.13	0.06	0.030
TJC	neg	low	0.13	0.03	1.8 × 10^–4^
	neg	high	0.13	0.04	1.9 × 10^–4^
	low	high	0.04	0.06	0.46
PtGA	neg	low	0.09	0.03	0.0081
	neg	high	0.06	0.04	0.097
	low	high	0.06	0.03	0.065
ESR	neg	low	0.09	0.03	0.0050
	neg	high	0.11	0.04	0.0024
	low	high	0.05	0.06	0.39
HAQ	neg	low	0.06	0.03	0.10
	neg	high	0.10	0.04	0.0048
	low	high	0.08	0.06	0.18
Pain VAS	neg	low	0.06	0.03	0.065
	neg	high	0.07	0.04	0.053
	low	high	0.02	0.06	0.69
PhGA	neg	low	0.17	0.03	1.0 × 10^–6^
	neg	high	0.14	0.04	2.4 × 10^–4^
	low	high	0.00	0.06	0.96

^a^Data from 974 to 978 patients were available for analyses except 902 for CDAI, 949 for HAQ, 966 for Pain VAS, and 903 for PhGA.

^b^The slope coefficients (β) with their standard errors (SE), and *p* values corresponding to the three specified comparisons in the unadjusted analysis are presented. The cut-off between low and high positive ACarPA was 3× the upper limit of normal.

We also explored if adjusting for the ACPA levels (negative, low positive, and high positive) in place of the ACPA presence changed the results. However, all the associations were very similar with the two ACPA adjustments (Supplementary Table S5). Besides, we looked for the potential association of activity measures with the interactions between the ACarPA and the other two RA autoantibodies (anti-CCP and RF). These analyses showed no significant association with the two-level (ACarPA × anti-CCP or ACarPA × RF) or the three level interactions (ACarPA × anti-CCP × RF, Supplementary Table S6). Finally, we also explored if the associations of the activity measures with the ACarPA were modified by the patients’ classification (Supplementary Table S7). In these analyses, the associations were slightly decreased compared to without accounting for the RA classification (presented in Tables 2, 3 and 4), but they mantained the same pattern. Specifically, the three composite activity measures, and the SJC, TJC, and PhGA were significantly associated with the presence of ACarPA (all *p* < 0.003), whereas the ESR and the three patient-reported outcomes were not associated.

### Persistently increased disease activity

**Figure 2:**
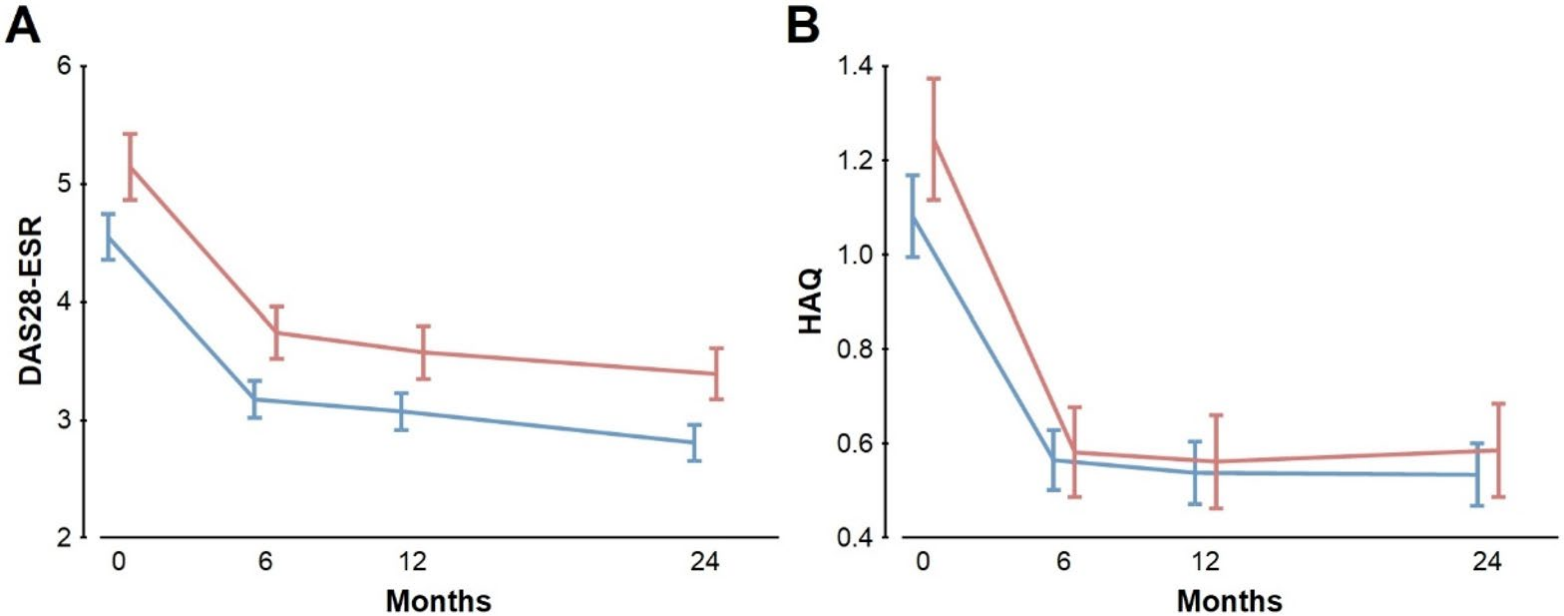
Persistent elevated DAS28-ESR but not HAQ in the EA ACarPA^+^ patients with complete follow-up data. (**A**) DAS28-ESR means and 95% CI in the 380 patients with data at the four follow-up times. Differences at each point *p* < 7.5 × 10^–4^. (**B**) HAQ means and 95% CI in the 434 patients with full follow-up data. Differences were not significant. The ACarPA^+^ patients are represented in red, whereas the ACarPA^−^ patients are in blue.

**Figure 3:**
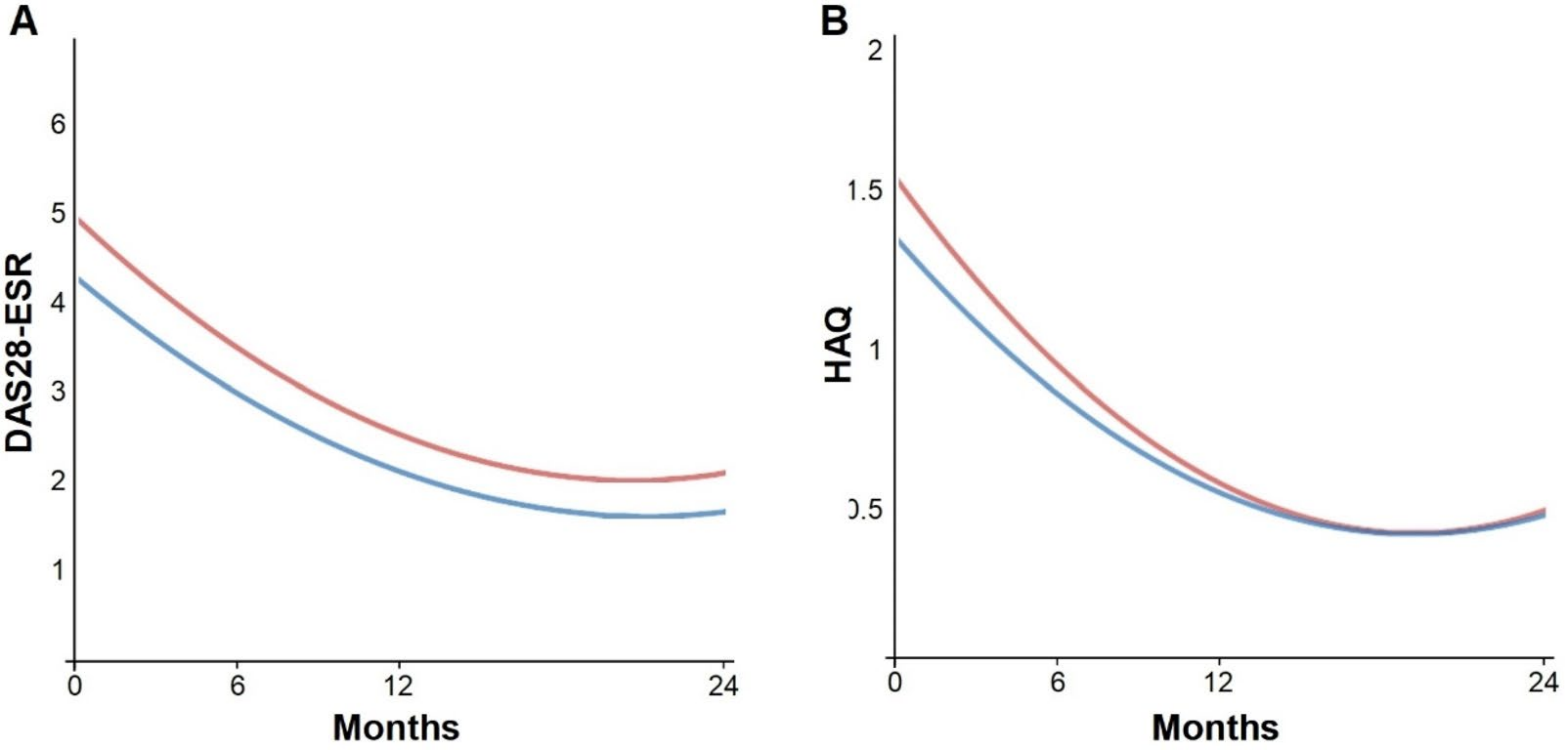
The overall estimated course of the DAS28-ESR and HAQ in the ACarPA^+^ and ACarPA^−^ patients considering all the available follow-up data. (**A**) The DAS28-ESR values were significantly different and the difference was constant, whereas the (B) HAQ was not different overall and showed a significant interaction with time. The ACarPA^+^ patients are represented in red, whereas the ACarPA^−^ patients are in blue.

The longitudinal analysis of the disease activity in the EA patients was done in two ways. First, we analyzed the subset of patients with complete data at the four follow-up visits (n = 380 for DAS28-ESR and 434 for HAQ, Fig. 2). Afterward, we analyzed all the patients irrespective of the missing follow-up data (Fig. 3; Table 6).

**Table 6:** Association of the DAS28-ESR and HAQ with ACarPA using all available follow-up data^a^.

Measure/Adjustment	Estimate (B)^b^	SE	p	Time interaction p
DAS28-ESR				
no^c^	0.46	0.10	1.6 × 10^–6^	0.13
no_crl_^c^	0.42	0.10	1.5 × 10^–5^	0.19
basic^c^	0.45	0.10	2.3 × 10^–6^	0.15
basic_crl_^c^	0.38	0.09	5.3 × 10^–5^	0.18
basic + CCP & RF^c^	0.28	0.10	5.2 × 10^–3^	0.17
basic + CCP & RF_crl_^c^	0.28	0.10	6.4 × 10^–3^	0.18
HAQ				
no^c^	0.03	0.04	0.47	0.022
basic + CCP & RF^c^	0.02	0.05	0.61	0.031

^a^Analyzed with mixed effects pattern mixture models including all available follow-up data for DAS28-ESR or HAQ, respectively.

^b^The estimated (B) overall increase in the activity measure in ACarPA^+^ relative to ACarPA^−^ patients, its standard error (SE) and *p* value are presented, as well as, the *p* values for the ACarPA status interaction with time.

^c^The analyses were performed as in Table 2 except when controlled (_crl_) by the inclusion of the two patterns showing MNAR data.

The patients with complete data showed a marked improvement in DAS28-ESR and HAQ from the first to the 6-month visit, with modest subsequent decreases (Table 1; Fig. 2). The initial improvement was such that it canceled the difference in HAQ between the ACarPA^+^ and ACarPA^−^ patients at all the follow-up visits (Fig. 2B). In contrast, the levels of DAS28-ESR continued to be elevated in the ACarPA^+^ patients (Fig. 2A). In effect, the ACarPA^+^ patients showed a higher DAS28-ESR than the ACarPA^−^ patients at all visits (*p* ≤ 7.5 × 10^–4^ at each visit). The DAS28-ESR difference decreased very slowly, from 0.59 (4.55 *vs.* 5.14) at the first visit to 0.56 (3.18 *vs.* 3.74), 0.50 (3.07 *vs.* 3.57) and 0.48 (2.91 *vs.* 3.39) at 6, 12 and 24 months, respectively (Fig. 2A). In other words, the DAS28-ESR values in the ACarPA^+^ and ACarPA^−^ patients showed an almost parallel evolution.

In the second type of analysis, we considered all the available follow-up information following the mixed-effects pattern-mixture model^42–44^. The results replicated the observed in the patients with complete follow-up (Fig. 3). On the one hand, there was a persistent increase in the DAS28-ESR along the study (Fig. 3A). On the other hand, there was a loss of the initial HAQ difference with time (Fig. 3B). However, in contrast to the visit-specific results reported in the preceding paragraph, only a set of parameters is obtained for the whole follow-up in this type of analysis. Hence, the DAS28-ESR differences between the ACarPA^+^ and ACarPA^−^ patients were 0.46 in the unadjusted and 0.28 in the fully adjusted analyses, respectively (Table 6). These differences were significant (*p* ≤ 5.2 × 10^–3^) and constant during the follow-up (p for the interaction with time ≥ 0.13). It should be noted that we identified two patterns of DAS28-ESR missing data that showed evidence of MNAR (Supplementary Table S8). Therefore, we built additional mixed-effects models controlling for them. The results were only visibly modified in the analyses not adjusted for the other RA autoantibodies, indicating that the confounding factors were already controlling for this potential bias. The HAQ, in turn, showed a small and non-significant overall increase in the ACarPA^+^ patients (Table 6). A contributing factor for the lack of overall HAQ difference was the significant interaction with time that canceled the increase observed at the first visit (p for the interaction with time ≤ 0.031, Table 6).

Further analyses on the available DAS28-ESR follow-up data showed a larger fraction of EA remaining with high disease activity (DAS28-ESR > 5.1) all along the follow-up, with a 13.0% *vs.* 6.5% (*p* = 0.007) at 6-months and 11.4% *vs*. 4.4% (*p* = 0.003) at 2-years. Also, there was a lower fraction of ACarPA^+^ patients achieving remission (i.e., DAS28-ESR < 2.6) during follow-up. In more detail, the remission rates at 6 months were 22.8% *vs.* 35.2% (*p* = 0.0043, analysis restricted to the patients with DAS28-ESR > 3.2 at the first visit) in the ACarPA^+^ and ACarPA^−^ patients. The difference was still significant at 2-years (35.4% *vs.* 45.8%, *p* = 0.04, in the same patients).

## Discussion

Our results have clarified the role of the ACarPA as short-term prognostic biomarkers in EA patients. First, they have replicated the finding of increased DAS28-ESR in the ACarPA^+^ patients^18,22,24^. Also, they have shown an increase of much less studied measures of disease activity in the ACarPA^+^ patients. Another notable finding of our study has been the persistence of the specific increase in DAS28-ESR. Therefore, we think the ACarPA can be considered a biomarker of severe disease in the two years of follow-up reflecting mainly the local component (SJC and TJC) of the DAS28. In the same line, the SJC was particularly increased in patients with high ACarPA levels. We think it is also worth of mention that the SJC, TJC, DAS28-ESR and PhGA associations with ACarPA were independent of the other RA autoantibodies (*p* < 0.001) and of interactions with them. The systemic and patient-reported measures were, in contrast, less increased and less specific of ACarPA relative to the other RA autoantibodies.

Most previous studies, although of high quality and large sample size, were limited to DAS28-ESR or HAQ as the only measures of disease activity^18,22,23^. All these studies detected a significant increase in DAS28-ESR associated with the presence of ACarPA at the first visit^18,22,24^. The replication of this finding is of importance because of the exceptionality of such a large degree of reproducibility. To date, this includes 6573 patients from six patient cohorts (including the current study and three cohorts in Derksen et al.) that have shown a significant association in each of the cohorts. Three of these cohorts were of EA patients, including the current and two previous studies^18,22^, the other three cohorts were of early RA patients^24^. Also, the association of DAS28-ESR was independent of RA classification in our patients. Therefore, the association of the presence of ACarPA with increased DAS28-ESR is not limited by RA classification. This property is of value to fulfill the need to assess the prognosis of EA patients in whom a definite diagnosis cannot yet be reached^3,5^. Furthermore, the DAS28-ESR increase was statistically independent of the presence of the analyzed RA autoantibodies in our study and four of the five previous cohorts^22,24^. In the remaining cohort, this specific analysis was not reported^18^. In addition, our results have shown that other composite measures of disease activity (CDAI and HUPI) produce similar results to the obtained with DAS28-ESR.

Another notable aspect of our results has been the confirmation of the persistence of the increase in disease activity. This increase, assessed with DAS28-ESR, was unclear before because it was only reported in two of the previous cohorts and with some caveats^18,22^. In one of the cohorts, the first follow-up time was at three years and the significant increase disappeared in the multivariate analysis including RF and anti-CCP^18^. In the other study, the increase in DAS28-ESR was only reported in the overall 15-year follow-up analyzed as a whole, including the first and up to three subsequent visits^22^. Therefore, our results, showing a significant increase at each visit and in the overall 2-year follow-up independently of RF and anti-CCP, provide a much-needed piece of information to establish the ACarPA as biomarkers. In this regard, we should note that the increase in DAS28-ESR was independent of the initial treatment, which did not include bDMARDs, as we have already reported^46^.

Only two studies including a total of three cohorts had addressed the association of ACarPA with elevated HAQ. In one of the cohorts, the HAQ increase was observed both in the first visit and in the overall period of follow-up independently of the presence of anti-CCP^22^. In the other study, the increase was observed only in one of the two cohorts and this association disappeared in the analysis including RF and anti-CCP^23^. Our results fit in this background because the increase in HAQ was only clear at the first visit and disappeared thereafter. In addition, our study provides a framework to interpret the HAQ results because the weak association with ACarPA was shared by the three patient-reported measures of disease activity. Elaborating on these ideas, it is possible to propose a hierarchy of ACarPA associations from the local to the patient-reported measures. In this hierarchy, all the patient-reported measures showed a weaker association than the physical examination and laboratory measures. This is congruent with the nature of the ACarPA, a laboratory biomarker, and highlights the imperfect and complementary relationship between the different disease activity measures^30,47^.

The current results pertaining to the short-term together with the previous reports showing the association of the ACarPA with poor long-term outcomes^12,15–17,19–21^ support the hypothesis that the ACarPA contribute to RA pathogenesis. The mechanism could be through increased inflammation via the formation of immune complexes that bind IgG Fc receptors and induce complement activation^48^. We think the specific association of increased SJC with high levels of ACarPA could be interpreted as resulting for this mechanism. A mechanism that does not exclude direct effects of the ACarPA on cells or proteins able to modulate joint inflammation.

Our study has some limitations. One that is common to all the ACarPA studies done to date is the unavailability of a standardized test. However, our homemade test has shown results comparable to the obtained by other laboratories^15,49,50^. A second limitation is that some of the patients were recruited more than a decade ago when standards of patient care were not according to current recommendations. However, the patients whose first visit took place since 2010 showed a similar increase of DAS28-ESR with the presence of ACarPA at each visit than the whole EA cohort (not shown). A third limitation is the evidence of MNAR data for DAS28-ESR. This circumstance calls for prudence in interpreting the follow-up results as representative of all the EA patients. However, it seems likely that the missing data were of little consequence according to our analyses controlling for them and because the underrepresented patients showed characteristics not associated with poor prognosis (low initial DAS28-ESR and low frequency of autoantibodies and RA diagnosis, Supplementary Table S8).

In conclusion, the ACarPA showed a notable and reproducible association with increased disease activity in EA patients. The reproducibility of this association is difficult to overemphasize. Especially, because the association with increased DAS28-ESR is reproducibly independent of the other RA autoantibodies and has been observed in EA and early RA cohorts. In addition, the increase has shown a dominance of the local component over the systemic and patient-reported components, and a notable persistence of the DAS28-ESR increase during follow-up. Further efforts should be directed to identify algorithms or patient subgroups in which the reproducible association could translate into clinical utility for the individual patient.

## Supplementary Information

Supplementary Information.
