# Supplementary material for: Increased disease activity in early arthritis patients with anti-carbamylated protein antibodies
Source: Sci Rep. 2021 May 11;11:9945. doi: 10.1038/s41598-021-89502-y (PMC8113595; doi:10.1038/s41598-021-89502-y)
Supplement: Supplementary file 1 — Supplementary Information. [file 41598_2021_89502_MOESM1_ESM.pdf]

Supplementary Table S1: Reported MCID or MCII for the RA activity measures that we used for result intrepretation [a]

Measure	MCID	MCII (95 % CI)	Obs.	Ref. [b]
DAS28-ESR		-1.17 (-1.36, -0.87)		Ward 2015
		-1.2 (-1.22, -1.18)	overall	Alataha 2009
		-1.9 if $DAS28_{bas}$ > 5.1	level specific	Alataha 2009
		-1.1 if $DAS28_{bas}$ 3.2-5.1	level specific	Alataha 2009
		-1.0 if l$DAS28_{bas}$ < 3,2	level specific	Alataha 2009
CDAI		-12.5 (-14.7, -10.5)		Ward 2015
		-10.76 (-11.04, -10.49)	overall	Alataha 2009
		-17.8 if $CDAI_{bas}$ > 22	level specific	Alataha 2009
		-7.3 if $CDAI_{ba}$s 10-22	level specific	Alataha 2009
		-1.8 if $CDAI_{bas}$ < 10	level specific	Alataha 2009
	12 if $CDAI_{bas}$ > 22		level specific	Curtis 2015
	6 if $CDAI_{bas}$ 10-22		level specific	Curtis 2015
	1 if $CDAI_{bas}$ < 10		level specific	Curtis 2015
HUPI		2		Gonzalez-Alvaro 2016
SJC	-	-		
TJC	-	-		
ESR	-	-		
PtGA		-18.4 (-26.0, -12.0)		Ward 2015
		-20		Kvamme 2010
HAQ		-0.375 (-0.5, -0.25)		Ward 2015
		-0.25	MHAQ	Kvamme 2010
Pain VAS		-20.4 (-24.0, -17.1)		Ward 2015
		-19		Kvamme 2010
PhGA	-	-		

[a] There are other reported MCID or MCII, but they were obtained in older and smaller studies than the selected

[b] The full references are provided in the main text

Supplementary Table S2. Classification of the EA patients at the end of the 2-year follow-up [a]

Classification	N	%
Rheumatoid arthritis	505	51.6
Undiferentiated arthritis	187	19.1
Miscellanea [b]	176	18.0
Spondyloarthritis	73	7.5
Connective Tissue Diseases	37	3.8

[a] The absence of uniform criteria for reporting all diseases, i.e., some doctors reported CTD whereas others detailed each individual CTD, made it impossible a more detailed list
[b] This group includes microcrystalline arthritis, reactive arthritis, polymyalgia rheumatica, palindromic syndrome, RS3PE, IBD-associated arthritis, and osteoarthritis

Supplementary Table S3. Association [a] of the activity measures with the ACarPA titers at the first visit.

	ρ [b]	p-value	N
DAS28-ESR	0.17	3.9×10^{-8}	978
CDAI	0.13	5.4×10^{-5}	902
HUPI	0.18	8.0×10^{-9}	974
SJC	0.15	2.2×10^{-6}	978
TJC	0.15	4.4×10^{-6}	978
PtGA	0.07	0.022	977
ESR	0.15	4.2×10^{-6}	975
HAQ	0.07	0.042	949
Pain	0.06	0.056	966
PhGA	0.14	2.5×10^{-5}	903

[a] The non-parametric Spearman rank correlation was used

[b] The Spearman regression coefficient (ρ), p-value, and number of subjects analyzed are presented

Supplementary Table S4. Multivariate analysis [a] of the SJC association with the ACarPA levels

Measure	1st level [b]	2nd level	β	SE	p-value
SJC	neg	low	0.08	0.03	0.023
	neg	high	0.13	0.04	5.4×10^{-4}
	low	high	0.11	0.06	0.063

[a] Multivariate analysis including the confounding factors sex, age, cohort, presence of RF and anti-CCP
[b] The slope coefficients (β) with their standard errors (SE) and p-values corresponding to the three specified comparisons are presented. The cut-off between low and high positive ACarPA was 3 x the upper limit of the negative.

Supplementary Table S5. Preserved association of the activity measures with ACarPA status after adjusting for ACPA levels [a]

Measure	Adjustement [b]	β [c]	SE	p-value	$ACarPA^+$ EMM	$ACarPA^-$ EMM
DAS28-ESR	level	0.13	0.03	1.9 x10-4	4.74 (4.51-4.97)	4.29 (4.11-4.47)
	status	0.13	0.03	1.3 x10-4	4.71 (4.52-4.91)	4.26 (4.12-4.40)
CDAI	level	0.11	0.04	1.7 x10-3	22.0 (19.9-24.1)	18.6 (16.9-20.2)
	status	0.11	0.04	1.5 x10-3	21.7 (19.9-23.5)	18.3 (17.0-19.5)
HUPI	level	0.12	0.03	5.6 x10-4	7.88 (7.39-8.36)	7.00 (6.6-7.38)
	status	0.12	0.03	4.3 x10-4	7.81 (7.40 -8.22)	6.92 (6.63-7.21)
SJC	level	0.11	0.03	9.9 x10-4	4.64 (3.92-5.42)	3.45 (2.96-3.97)
	status	0.12	0.03	0.0008	4.49 (3.88-5.15)	3.31 (2.92-3.69)
TJC	level	0.11	0.03	1.0 x10-3	4.69 (3.82-5.65)	3.25 (2.68-3.87)
	status	0.12	0.03	0.0006	4.97 (4.20-5.81)	3.42 (2.96-3.92)
PtGA	level	0.07	0.04	0.040	48.3 (44.4-52.2)	44.1 (41.1-47.2)
	status	0.07	0.04	0.049	46.78 (43.47-50.08)	42.81 (40.49-45.13)
ESR	level	0.06	0.03	0.073	24.2 (21.3-27.5)	21.5 (19.5-23.8)
	status	0.06	0.03	0.067	23.57 (21.12-26.05)	20.91 (19.30-22.42)
HAQ	level	0.08	0.04	0.029	1.12 (1.00-1.23)	0.99 (0.90-1.08)
	status	0.08	0.04	0.02	1.13 (1.03-1.23)	0.99 (0.92-1.06)
Pain	level	0.06	0.04	0.097	49.1 (45.0-53.3)	45.6 (42.4-48.8)
	status	0.06	0.04	0.117	47.54 (44.02-51.05)	44.19 (41.72-46.65)
PhGA	level	0.15	0.04	4.0 x10-5	43.3 (39.8-46.7)	35.9 (33.2-38.6)
	status	0.15	0.04	3.98 x10-5	41.92 (38.99-44.85)	34.59 (32.52-36.67)

[a] The ACPA titers were classified as negative, low positive, and high positive. The threshold between low and high positive was 3 x the upper limit of the negative

[b] The analyses were adjusted by cohort, sex, age, presence of RF, and either the ACPA level (level) or the ACPA status (status). The results adjusted for status are already presented in Tables 2-4

[c] The standardized coefficients (β), its SE and p-value, and the estimated marginal means (EMM) with their 95 % CI are presented

Supplementary Table S6. Lack of significant association between the activity measures and the three ACarPA autoantibody interactions [a]

Measure	Interaction term [b]	β [c]	SE	p-value
DAS28-ESR	ACarPA x ACPA	0.02	0.04	0.71
	ACarPA x RF	-0.04	0.04	0.37
	ACarPA x ACPA x RF	0.01	0.04	0.81
CDAI	ACarPA x ACPA	0.02	0.04	0.66
	ACarPA x RF	-0.03	0.05	0.45
	ACarPA x ACPA x RF	-0.02	0.04	0.66
HUPI	ACarPA x ACPA	0.02	0.04	0.68
	ACarPA x RF	-0.05	0.04	0.24
	ACarPA x ACPA x RF	0.002	0.04	0.96
SJC	ACarPA x ACPA	0.03	0.04	0.43
	ACarPA x RF	-0.02	0.04	0.61
	ACarPA x ACPA x RF	-0.02	0.04	0.64
TJC	ACarPA x ACPA	0.03	0.04	0.55
	ACarPA x RF	-0.02	0.04	0.67
	ACarPA x ACPA x RF	-0.02	0.04	0.64
PtGA	ACarPA x ACPA	-0.02	0.04	0.65
	ACarPA x RF	-0.03	0.04	0.47
	ACarPA x ACPA x RF	0.007	0.04	0.87
ESR	ACarPA x ACPA	0.00	0.04	0.96
	ACarPA x RF	-0.06	0.04	0.15
	ACarPA x ACPA x RF	0.05	0.04	0.24
HAQ	ACarPA x ACPA	0.02	0.04	0.73
	ACarPA x RF	-0.02	0.04	0.62
	ACarPA x ACPA x RF	-0.01	0.04	0.79
Pain	ACarPA x ACPA	-0.04	0.04	0.37
	ACarPA x RF	-0.004	0.05	0.92
	ACarPA x ACPA x RF	0.02	0.04	0.73
PhGA	ACarPA x ACPA	-0.003	0.04	0.94
	ACarPA x RF	-0.04	0.05	0.37
	ACarPA x ACPA x RF	0.0005	0.04	0.99

[a] The three interaction terms including ACarPA were analyzed in the same model including the antibody main effects and the confounding variables

[b] The analyses were adjusted by cohort, sex, age, presence of RF, ACPA, and ACarPA.

[c] The standardized coefficients (β), its SE, and p-value are presented.

Supplementary Table S7. Association of the activity measures at the first visit with the ACarPA status accounting for the final patient classification.

Measure	Adjustment	β [a]	SE	p-value	$ACarPA^+$ EMM	$ACarPA^-$ EMM
DAS28-ESR	basic + anti-CCP & RF [b]	0.13	0.03	1.3×10^{-4}	4.71 (4.52-4.91)	4.26 (4.12-4.40)
	basic + anti-CCP & RF + RA/no [c]	0.11	0.03	8.3×10^{-4}	4.57 (4.38-4.76)	4.19 (4.06-4.32)
	basic + anti-CCP & RF + other Dx [d]	0.11	0.03	8.6×10^{-4}	4.34 (4.12-4.56)	3.97 (3.79-4.14)
CDAI	basic + anti-CCP & RF	0.11	0.04	1.5×10^{-3}	21.7 (19.9-23.5)	18.3 (17.0-19.5)
	basic + anti-CCP & RF + RA/no	0.09	0.03	6.0×10^{-3}	20.3 (18.6-22.0)	17.5 (16.3-18.7)
HUPI	basic + anti-CCP & RF	0.12	0.03	4.3×10^{-4}	7.81 (7.40 -8.22)	6.92 (6.63-7.21)
	basic + anti-CCP & RF + RA/no	0.10	0.03	2.5×10^{-3}	7.49 (7.09-7.88)	6.76 (6.49-7.04)
SJC	basic + anti-CCP & RF	0.12	0.03	7.6×10^{-4}	4.49 (3.88-5.15)	3.31 (2.92-3.69)
	basic + anti-CCP & RF + RA/no	0.09	0.03	4.8×10^{-3}	3.98 (3.45-4.56)	3.09 (2.76-3.44)
TJC	basic + anti-CCP & RF	0.12	0.03	5.8×10^{-4}	4.97 (4.20-5.81)	3.42 (2.96-3.92)
	basic + anti-CCP & RF + RA/no	0.10	0.03	2.7×10^{-3}	4.47 (3.76-5.24)	3.22 (2.80-3.68)
PtGA	basic + anti-CCP & RF	0.07	0.04	0.049	46.8 (43.5-50.1)	42.8 (40.5-45.1)
	basic + anti-CCP & RF + RA/no	0.06	0.03	0.10	45.4 (42.1-48.7)	42.1 (39.8-44.4)
ESR	basic + anti-CCP & RF	0.06	0.03	0.067	23.6 (21.1-26.1)	20.9 (19.3-22.4)
	basic + anti-CCP & RF + RA/no	0.05	0.03	0.13	22.6 (20.3-25.1)	20.4 (19.0-22.0)
HAQ	basic + anti-CCP & RF	0.08	0.04	0.02	1.13 (1.03-1.23)	0.99 (0.92-1.06)
	basic + anti-CCP & RF + RA/no	0.06	0.03	0.061	1.07 (0.98-1.17)	0.98 (0.90-1.03)
Pain	basic + anti-CCP & RF	0.06	0.04	0.12	47.5 (44.0-51.1)	44.2 (41.7-46.7)
	basic + anti-CCP & RF + RA/no	0.05	0.03	0.20	46.4 (42.9-49.9)	43.6 (41.2-46.1)
PhGA	basic + anti-CCP & RF	0.15	0.04	3.98×10^{-5}	41.9 (39.0-44.8)	34.6 (32.5-36.7)
	basic + anti-CCP & RF + RA/no	0.13	0.03	2.0×10^{-4}	39.8 (37.0-42.6)	33.4 (31.4-35.6)

[a] The slope coefficients (β) with their standard errors (SE), and p-values, and the estimated marginal means (EMM) with their 95 % CI are presented

[b] The cohort, gender, age, RF status and anti-CCP status were included as confounding variables. These results are also presented in tables 2-4 and are reported here for comparison.

[c] The cohort, gender, age, RF status, anti-CCP status, and RA/non RA class were included as confounding variables. The patients were classified according to the 1987 ACR classification criteria at the end of the 2-year follow-up.

[d] The cohort, gender, age, RF status, anti-CCP status, and the classification of patients in the 5 categories of Table S2 were included as confounding variables

Supplementary Table S8. Patterns of missing data in the follow-up, the MNAR evidence for each [a], and other features [b]

		Month					1st visit				1st visit features			
	Pattern #	0	6	12	24	N	DAS28-ESR mean (SD)	HAQ mean (SD)	p	Meaning	ACarPA %	anti-CCP %	RF %	RA class at 2-years %
DAS28-ESR	1					56	4.24 (1.40)		0.58		37.5	42.9	50.0	60.7
	2					38	4.16 (1.52)		0.75		34.2	47.4	50.0	57.9
	3					65	4.73 (1.67)		0.18		33.8	50.8	53.8	70.8
	4					29	4.58 (1.59)		0.98		24.1	37.9	34.5	41.4
	5					18	4.39 (1.36)		0.73		33.3	44.4	55.6	66.7
	6					154	4.27 (1.28)		5.7×10^{-4}	MNAR	20.8	22.7	27.3	18.2
	7					238	4.23 (1.56)		1.9×10^{-3}	MNAR	20.6	18.5	24.8	23.9
	8					380	4.75 (1.60)		NA		32.9	53.4	56.1	77.4
HAQ	1					29		NA	NA		31.0	20.7	44.8	31.0
	2					54		1.05 (0.67)	0.47		38.9	35.2	40.3	55.6
	3					42		1.00 (0.74)	0.67		28.6	35.7	45.2	45.2
	4					102		1.08 (0.79)	0.98		28.4	41.2	40.2	49.0
	5					28		1.23 (0.80)	0.09		28.6	35.7	32.1	39.2
	6					20		1.02 (0.68)	0.14		10.0	30.0	35.0	30.0
	7					77		0.99 (0.76)	0.33		28.6	28.6	33.8	33.8
	8					192		0.98 (0.74)	0.11		19.8	19.8	23.4	22.9
	9					434		1.13 (0.76)	NA		30.9	50.2	53.2	71.4

[a] The colored cells are missing data. N = number; p of each pattern association with either DAS28-ESR or HAQ; NMAR = Not Missing At Random.

[b] The fraction of patients with antibody status at the first visit or RA classification at the end of the study in each of the missing data patterns

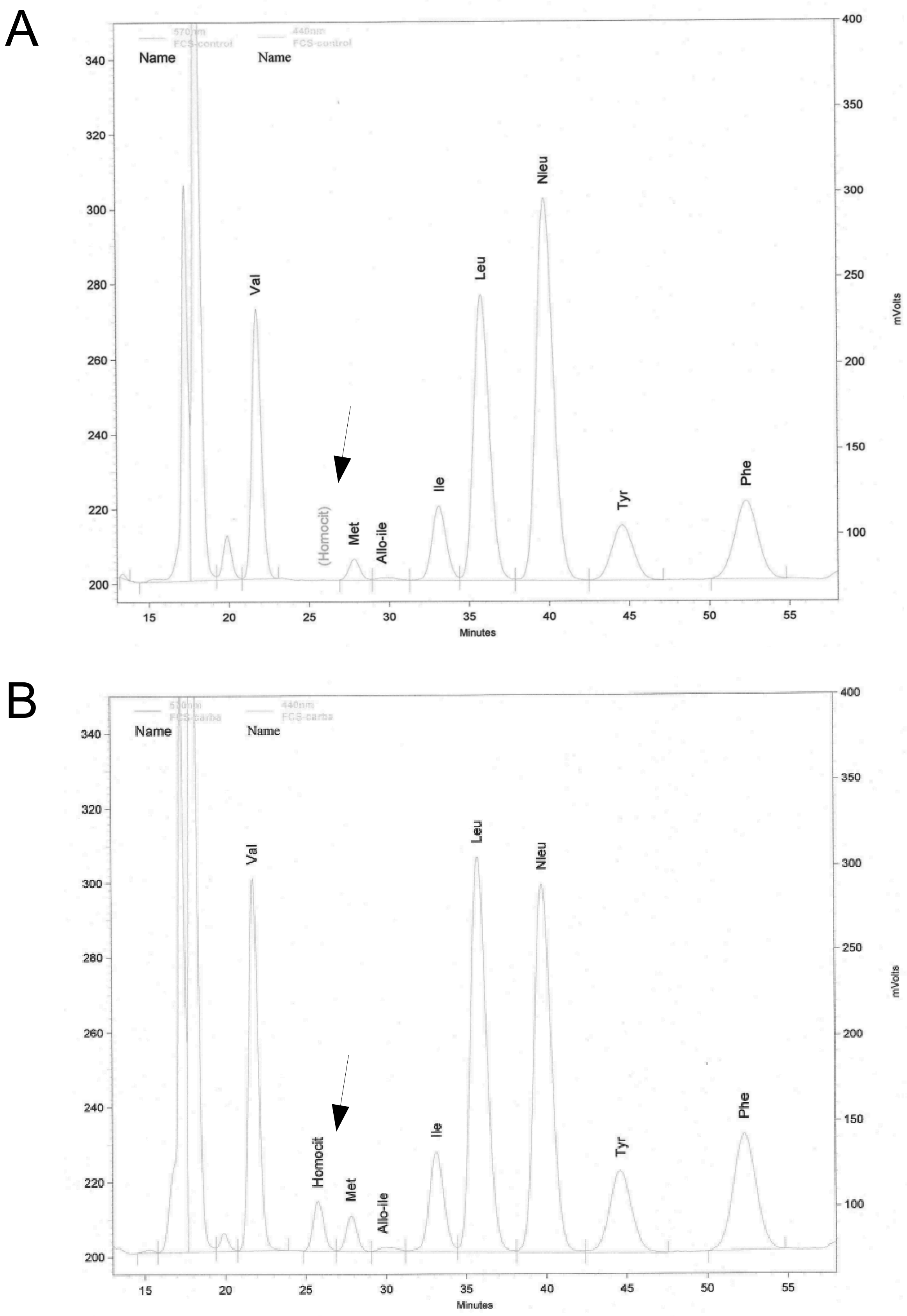

Supplementary Fig. S1. Chromatogram showing the separation of digested amino acids from A) unmodified FCS, and B) in vitro carbamylated FCS. The two antigens were used for ELISA in the detection of ACarPA. The focus was placed on homocitrulline (arrows) and the spiked L-Norleucine standard. Lysine runs at later times.

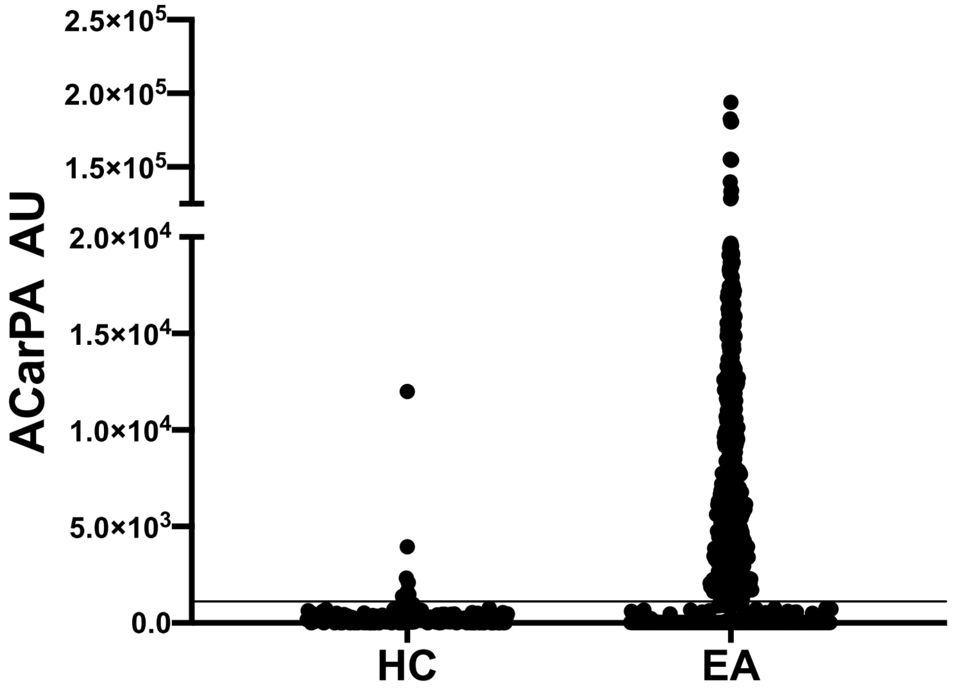

Supplementary Fig. 2. Level of ACarPA in EA patients and healthy controls. The ACarPA arbitrary units (AU) in 208 healthy controls and the 978 early arthritis (EA) patients are represented. Each dot corresponds to a single patient. The horizontal line signals the cut-off distinguishing ACarPA negative and positive subjects.

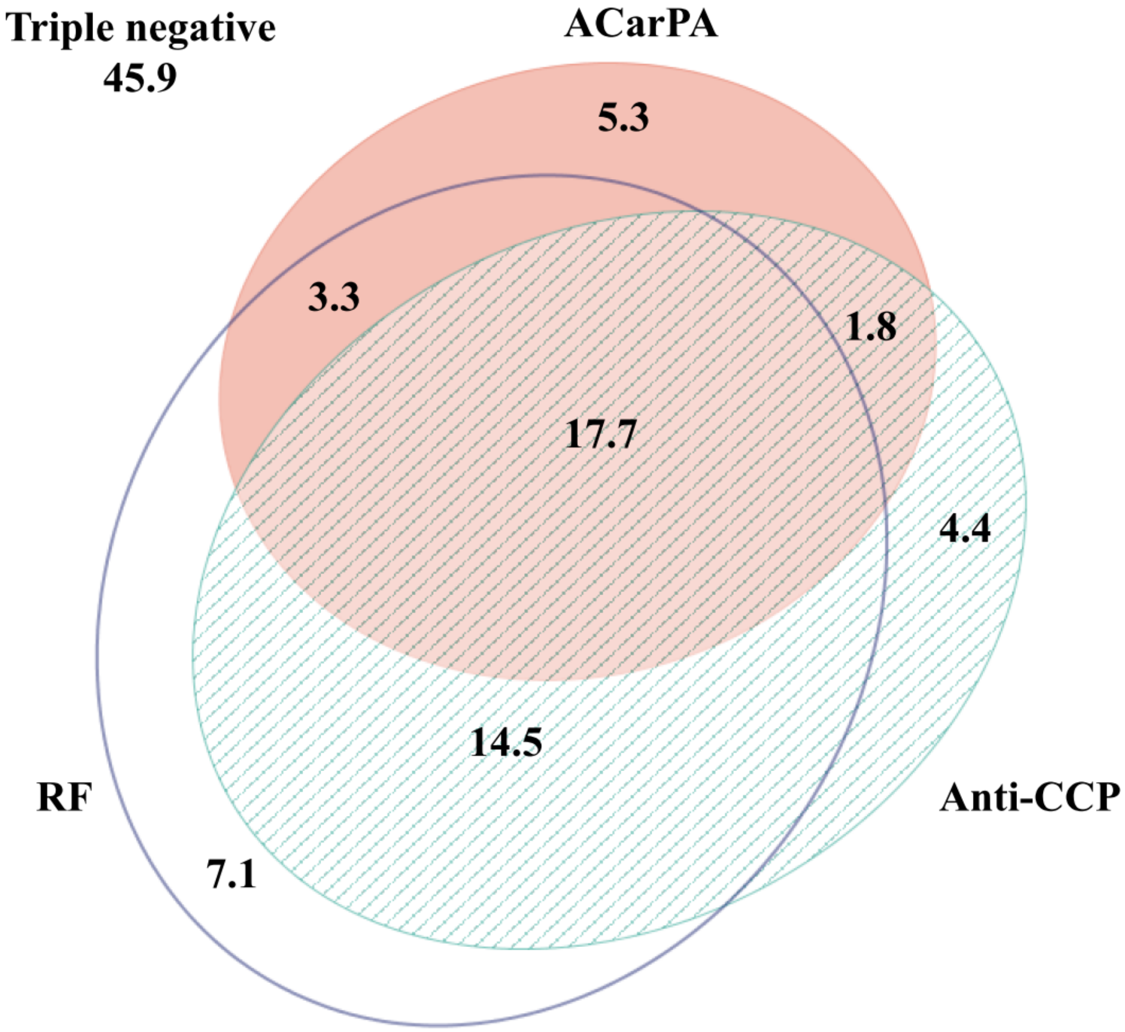

Supplementary Fig. S3: Proportional Venn diagram showing the distribution of the three RA autoantibodies in the EA patients. All the numbers are % of the total.
